# Effects of *Securigera securidaca* Extract on Lipolysis and Adipogenesis in Diabetic Rats

**DOI:** 10.1155/2014/582106

**Published:** 2014-08-03

**Authors:** Ahmad Ghorbani, Reyhaneh Moradi Marjaneh, Ziba Rajaei, Mousa-Al-Reza Hadjzadeh

**Affiliations:** ^1^Pharmacological Research Center of Medicinal Plants, School of Medicine, Mashhad University of Medical Sciences, Mashhad 9177948564, Iran; ^2^Neurocognitive Research Center and Department of Physiology, School of Medicine, Mashhad University of Medical Sciences, Mashhad 9177948564, Iran; ^3^Department of Physiology, School of Medicine, Isfahan University of Medical Sciences, Isfahan 7346181746, Iran

## Abstract

Diabetes mellitus is associated with dysregulation of adipose tissue metabolism and increased level of serum lipids. In our previous work we found that *Securigera securidaca* decreases cholesterol level in blood of diabetic rats. The present study was carried out to further investigate the effects of this plant on lipid metabolism, lipolysis, and adipogenesis, in diabetic rats. Female Wistar rats were rendered diabetic by intraperitoneal injection of streptozotocin. Retroperitoneal adipose tissue was removed from diabetic animals after seven days of streptozotocin injection. Effect of hydroalcoholic extract of *S. securidaca* seeds (100–800 *μ*g/mL) on adipose tissue lipolysis was evaluated in *ex vivo* condition. Also, to evaluate adipogenesis, preadipocytes were isolated from adipose tissue and differentiated to adipocytes in the presence of the extract. The extract at concentration of 800 *μ*g/mL decreased both basal and catecholamine-stimulated lipolysis (*P* < 0.05). Incubation of differentiating preadipocytes with 800 *μ*g/mL of *S. securidaca* extract decreased intracellular lipid droplet accumulation as evaluated with Oil Red O staining (*P* < 0.001). The extract even at high concentrations had no effect on viability of preadipocytes. In conclusion, *S. securidaca* decreases lipolysis and adipogenesis without cytotoxicity, which makes it a good candidate for management of dyslipidemia and reduction of cardiovascular risks in diabetes.

## 1. Introduction

Diabetes mellitus is a major cause of hospitalization and still is one of the main diseases causing death and disability. The number of diabetic patients is markedly increasing in the world. According to the World Health Organization reports (October, 2013), 347 million people suffer from diabetes worldwide and without urgent action, it will be the 7th cause of mortality in 2030. Diabetes is associated with impaired glucose and lipid metabolism and over time leads to microvascular and macrovascular complications such as cardiovascular diseases [1]. Dyslipidemia, a main risk factor of cardiovascular diseases, is often present in diabetic patients. Diabetic dyslipidemia is characterized by increased serum triglyceride and low density lipoprotein and decreased high density lipoprotein [2]. Patients with type-1 diabetes also undergo dysregulation of adipose tissue metabolism (lipolysis and lipogenesis) due to insulin deficiency.

Currently, statins, fibrates, niacin, and bile acid binding sequestrants are the most widely used medications for dyslipidemia. However, the clinical uses of these drugs are accompanied with unpleasant side effects such as myopathy and hepatic toxicity [3, 4]. Moreover, despite aggressive drug therapy, a number of diabetic patients still experience coronary heart disease events [5]. Therefore, finding new hypolipidemic agents with better efficacy and lesser side effects is promising approach for management of diabetic dyslipidemia.

Several studies have shown beneficial effects of natural agents on diabetes associated hyperglycemia and dyslipidemia [6, 7].* Securigera securidaca*, an annual herb belonging to the Fabaceae family, is used in Iranian folk medicine for treatment of diabetes. Experimental studies have revealed that administration of* S. securidaca* seeds decreases blood glucose in normal and diabetic subjects [8–10]. Also this plant reduces the level of triglyceride and cholesterol in serum of high-fat fed rats [11]. In our previous work we showed that hydroalcoholic extract of* Securigera securidaca* seeds decreases serum cholesterol in diabetic rats [12]. This study was carried out to investigate effects of this extract on adipose tissue lipolysis and adipogenesis in diabetic rats.

## 2. Materials and Methods

### 2.1. Chemicals and Reagents

Dulbecco's modified Eagle's medium (DMEM) and fetal bovine serum (FBS) were purchased from Gibco (Carlsbad, CA). Streptozotocin (STZ) was obtained from Enzo Life Science (USA). Fatty acid-free bovine serum albumin fraction V, glycerol assay reagent, isoproterenol, penicillin-streptomycin, type-II collagenase, 3-(4,5-dimethyl-2-thiazolyl)-2,5-diphenyl-2H-tetrazolium bromide (MTT), and 4-(2-hydroxyethyl) piperazine-1-ethanesulfonic acid sodium salt (HEPES) were provided from Sigma (USA). Dimethyl sulfoxide and 3-isobutyl-1-methylxanthine (IBMX) were purchased from Fluka Chemical Co. Indomethacin and human insulin were kindly provided by EXIR Company (Iran).

### 2.2. Preparation of Extract

The* S. securidaca* seeds were powdered with a blender and 860 g of the powder was suspended in 3200 mL of 70% ethanol. The mixture was left in dark at room temperature for 72 h under gentle shaking. The hydroalcoholic extract was then filtered and dried on a water bath.

### 2.3. Animals

Female albino Wistar rats (230 ± 30 g) were housed in a room with controlled lighting (12 h light/12 h darkness) and temperature (22 ± 2°C). All animal studies were carried out in accordance with the ethical guidelines of the animal care of the Mashhad University of Medical Sciences, Iran. Diabetes was induced by a single intraperitoneal injection of STZ (50 mg/kg). Three days after STZ injection, induction of diabetes was confirmed by measuring fasting blood glucose (FBG). Animals were considered to be diabetic if they had FBG of 250 mg/dL or higher [13, 14].

### 2.4. Lipolysis Studies

The effect of* S. securidaca* extract on adipose tissue lipolysis was evaluated in* ex vivo* condition [15, 16]. Retroperitoneal adipose tissues were removed from diabetic animals after seven days of STZ injection. The tissues were minced into uniform small slices of about 5 mg. The tissue slices were washed with phosphate-buffered saline, dried on the gauze, and weighted precisely. Then, tissue slices were distributed into 24-well plate (100 mg/well) and bathed with 1 mL Krebs-Ringer bicarbonate buffer supplemented with 25 mM HEPES, 5.5 mM glucose, and 2% (w/v) bovine serum albumin. The tissues were treated with vehicle (basal lipolysis) or isoproterenol (stimulated lipolysis) in the absence or presence of* S. securidaca* extract under constant shaking for 90 min at 37°C. At the end of the treatment, glycerol concentration was measured in the buffer by an enzymatic method.

### 2.5. Preadipocyte Preparation and Adipogenesis Assay

On day seven of diabetes inception, the rats were anesthetized with ether and retroperitoneal adipose tissue was excised through a sterile laparotomy procedure. The tissue sample was sliced into small pieces and washed with phosphate-buffered saline (PBS). The tissue pieces were then digested in PBS containing 2 mg/mL collagenase under shaking (60 cycles/min) at 37°C. The cellular pellet was isolated via centrifugation (2000 rpm for 5 min) and suspended in DMEM medium supplemented with 10% FBS, 100 units/mL penicillin, and 100 *μ*g/mL streptomycin. The cells were plated in culture flask and incubated in a humidified 5% CO_2_ incubator until they reached confluence. The cells were then trypsinized and seeded in 12-well plates (10^4^ cells/well). After 24 h of incubation, the culture medium was changed into differentiation medium (DMEM supplemented with 3% FBS, 250 *μ*M IBMX, 34 *μ*M d-pantothenate, 1 *μ*M dexamethasone, 0.2 *μ*M insulin, and 5 *μ*M indomethacin). After 3 days, the cells were exposed to the adipocyte maintenance medium (DMEM supplemented with 3% FBS, 34 *μ*M d-pantothenate, 1 *μ*M dexamethasone, and 0.2 *μ*M insulin). The cells were further cultured in this medium for 6 days and the medium was changed every 3 days [17]. To evaluate the effects of* S. securidaca* extract on adipogenesis, both differentiation and adipocyte maintenance media were supplemented with varying concentrations of the extract or vehicle (1% DMSO).

### 2.6. Oil Red O Staining

To examine the effect of* S. securidaca* on adipogenesis, Oil Red O was used to stain accumulated intracellular triglycerides in differentiated adipocytes. After 9 days of differentiation, the cells were fixed using 10% formalin and then stained by 200 *μ*L Oil Red O solution. After three times washing with distilled water, the stain was eluted from cells using 200 *μ*L isopropanol and its optical density was read at 545 nm [18, 19].

### 2.7. Cell Viability Assay

Effect of* S. securidaca* extract on viability of isolated rat preadipocytes and L929 mouse fibroblast cells was determined using MTT colorimetric assay. The cells were seeded (5000/well) in 96-well culture plates containing DMEM medium supplemented with antibiotic and 10% FBS. After 24 h, the medium was changed to a fresh one containing various concentrations of* S. securidaca* extract and the cells were further incubated for 48 h. At the end of incubation, the MTT solution was added to each well at final concentration of 0.5 mg/mL and the plate was placed for 2 h at 37°C [20, 21]. Then, the supernatant was discarded and the resulting formazan was dissolved by adding 200 *μ*L dimethyl sulfoxide to each well. The absorbance of formazan dye was read at 545 nm against 630 nm as background.

### 2.8. Statistical Analysis

The values were compared using the one-way analysis of variance followed by Tukey's post hoc test. *P* value < 0.05 was considered to be statistically significant. The results are presented as mean ± SEM.

## 3. Results

### 3.1. Effect of* S. securidaca* on Lipolysis

**Figure 1:**
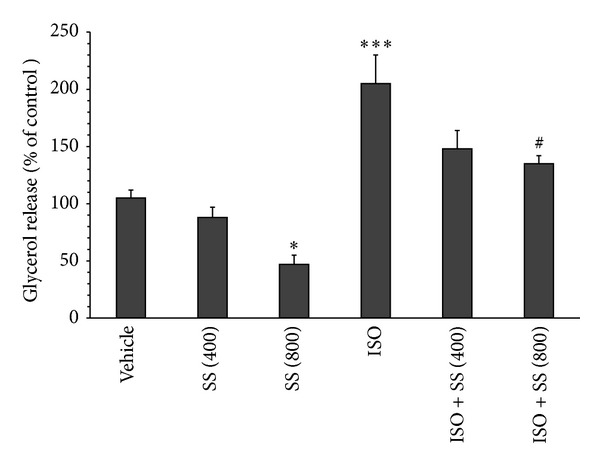
Effects of hydroalcoholic extract of* Securigera securidaca* on lipolysis in diabetic rats. Retroperitoneal adipose tissues were treated with vehicle or* Securigera securidaca* (SS) in the absence (basal lipolysis) or presence of 1 *μ*M isoproterenol (ISO) for 90 min. Concentration of SS is shown in parentheses (*μ*g/mL). Data are presented as means ± SEM of 6 independent experiments. *P < 0.05 versus vehicle; ***P < 0.001 versus control; ^#^ *P* < 0.05 versus ISO.

Figure 1 demonstrates the effect of* S. securidaca* extract on basal and ISO-stimulated lipolysis. In basal condition, the extract at concentration of 800 *μ*g/mL significantly decreased glycerol release to 47 ± 8% of control (*P* < 0.05). To examine the effect of* S. securidaca* on stimulated lipolysis, the lipolytic activity was tested in the presence of isoproterenol, a nonselective beta adrenergic receptor agonist. As expected, 1 *μ*M isoproterenol led to significant elevation in glycerol release (205 ± 25% of control, *P* < 0.001). The extract at concentration of 800 *μ*g/mL significantly reduced the stimulated lipolysis to 135 ± 7% of control (*P* < 0.05).

### 3.2. Effects of* S. securidaca* on Adipogenesis

**Figure 2:**
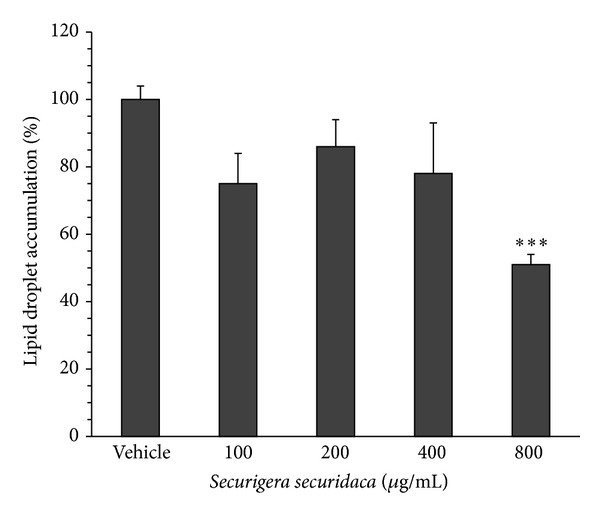
Effect of* Securigera securidaca* on lipid droplet accumulation in differentiating preadipocyte isolated from diabetic rats. The lipid accumulation was estimated by measuring the optical density of Oil Red O stain eluted from cells. Data are mean ± SEM (*n* = 5). *P < 0.001 versus vehicle.

Incubation of differentiating cells with* S. securidaca* extract decreased intracellular lipid droplet accumulation as evaluated with Oil Red O staining (Figure 2). The presence of 100, 200, 400, and 800 *μ*g/mL of the extract in the culture medium decreased the lipid droplet content from 100 ± 4% (untreated cells) to 75 ± 9%, 86 ± 8%, 78 ± 15%, and 51 ± 3% (*P* < 0.001), respectively.

### 3.3. Effect of* S. securidaca* on Viability of Preadipocytes

**Figure 3:**
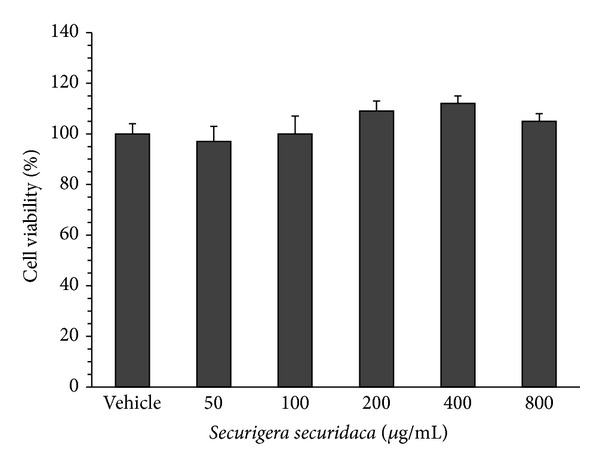
Effect of* Securigera securidaca* on viability of preadipocytes isolated from diabetic rats. The cells were cultured in the presence of hydroalcoholic extract of* S. securidaca* for 24 h. The bars show percent of cell viability as compared with untreated cells (vehicle). Data are mean ± SEM (*n* = 8).

None of* S. securidaca* concentrations decreased proliferation of preadipocytes. In the presence of 50, 100, 200, 400, and 800 *μ*g/mL of* S. securidaca* extract, viability of the cells was 97 ± 6%, 100 ± 7%, 109 ± 4%, 112 ± 3%, and 105 ± 3%, respectively (Figure 3).

## 4. Discussion

Dysregulation of lipid metabolism is a key feature of some pathological conditions including diabetes mellitus, insulin resistance, obesity, and fatty liver [22, 23]. In diabetes, a spectrum of abnormalities including increased serum lipids, uncontrolled lipolysis, and dysregulation of adipogenesis and lipogenesis was involved in development of atherosclerosis and cardiovascular diseases [2, 24]. It has been shown that* S. securidaca* reduces the serum level of triglyceride and cholesterol in hypercholesterolemic animals [11]. Also, in our previous work we showed that hydroalcoholic extract of this plant decreases serum cholesterol in diabetic rats [12]. In the present study, we further evaluated the effects of* S. securidaca* on adipose tissue in STZ-induced diabetes. Our data showed that this extract significantly decreases adipocyte lipolysis and differentiation of preadipocytes.

It has been well documented that hypercholesterolemia is associated with an increase in risks of atherogenesis in diabetes mellitus [2, 25]. Because of hypocholesterolemic action, consumption of* S. securidaca* may improve the management of diabetic dyslipidemia and reduce the incidence of cardiovascular events in diabetic patients. Atherogenic dyslipidemia is caused by different metabolic abnormalities including (1) increased cholesterol synthesis, (2) increased production of triglyceride-rich lipoproteins, and (3) increased HDL catabolism. It is believed that among these abnormalities, the pivotal role is played by increased hepatic production of lipoproteins [2]. Availability of triglycerides within hepatocytes is an important factor influencing synthesis of very low density lipoprotein (VLDL) [26]. Triglycerides are provided from* de novo* synthesized or extrahepatic fatty acids. Adipose tissue-derived free fatty acid is the largest extrahepatic source of fatty acid for triglyceride synthesis [2, 27]. Fatty acids are released from adipose tissue through a highly regulated process named lipolysis. Although a variety of factors are involved in regulation of lipolysis, in physiologic situation insulin and catecholamines are the main antilipolytic and prolipolytic agents, respectively [28]. Our results showed that* S. securidaca* extract inhibits both basal and catecholamine-stimulated lipolysis. Therefore, the antilipolytic action of* S. securidaca* may be responsible in part for its effect in decreasing blood cholesterol. In diabetes, deficiency of insulin in conjunction with glucagon- or catecholamine-stimulated lipolysis increases fatty acid delivery to liver which may lead to ketoacidosis, a life-threatening condition [29]. It is reasonable to conclude that* S. securidaca* can decrease the risk of ketoacidosis through inhibition of lipolysis. One limitation of our study is that we could not test more concentrations of* S. securidaca* on lipolysis because a limited amount of adipose tissue can be obtained from diabetic rats (the animals show rigorous weight loss).

The mass of adipose tissue is determined by the number and size of adipocytes. Number of adipocytes is dependent on the rate of formation of new adipocytes from precursor cells (adipogenesis) and the rate of adipocyte apoptosis. Size of adipocytes is increased by lipogenesis and decreased by lipolysis [30]. Our data showed that* S. securidaca* extract inhibits differentiation of preadipocyte to adipocyte. This effect in conjunction with its antilipolytic action makes* S. securidaca* a good candidate for management of diabetic dyslipidemia in obese diabetic patients. Because the* S. securidaca* extract had no effect on viability of preadipocytes, most probably its beneficial actions on lipid metabolism are not accompanied by cytotoxic effect.

In conclusion, the present study demonstrated that extract of* S. securidaca* seed inhibits lipolysis and adipogenesis in diabetic animals. These effects make this plant a good candidate for management of diabetic dyslipidemia and reduction of cardiovascular risk in diabetic patients.
